# HALP score in *Demodex* blepharitis: A case–control study

**DOI:** 10.1515/med-2025-1257

**Published:** 2025-08-22

**Authors:** Nuri Cakir, Emine Pangal, Isil Cakir, Ozan Yaman, Nurettin Bayram

**Affiliations:** Clinical Microbiology and Immunology, Bunyan State Hospital, Kayseri, 38000, Turkey; Clinical Ophthalmology, Kayseri City Training and Research Hospital, Kayseri, 38000, Turkey; Clinical Biochemistry, Kayseri City Training and Research Hospital, Kayseri, 38000, Turkey; Clinical Parasitology, Kayseri City Training and Research Hospital, Kayseri, 38000, Turkey; Clinical Ophthalmology, Ankara Etlik City Training and Research Hospital, Ankara Etlik, Ankara, 06000, Turkey

**Keywords:** *Demodex* blepharitis, inflammation, HALP score, pan, immune, inflammation value, systemic immune inflammation index

## Abstract

**Objectives:**

*Demodex* mite infestation is one of the most prevalent causes of blepharitis. This study was designed to evaluate whether *Demodex* blepharitis was related to novel inflammatory markers.

**Methods:**

89 patients with *Demodex* blepharitis and 76 age-matched participants without blepharitis enrolled in the study. Test parameters such as hemoglobin, albumin, neutrophil, lymphocyte, monocyte, platelet, WBC, CRP, HALP score, systemic immune-inflammation-index (SII), pan-immune-inflammation value (PIV), and *Demodex* density were evaluated.

**Results:**

CRP values were numerically higher, and albumin levels were lower in the patient group, even though the differences between these levels in both groups were not statistically significant (*p* > 0.05). SII and PIV indices were shown to be numerically higher, and HALP score levels were statistically significantly lower in the patient group (*p* = 0.134, *p* = 0.319, *p* = 0.001). The ROC analysis was carried out, and the optimal cutoff point for the HALP score was designated using the formula of Youden’s index. It was suggested that values below 504.8 for the HALP score can be used in the diagnosis of *Demodex* blepharitis with 66.5% of sensitivity and 78% of specificity.

**Conclusions:**

CRP, SII, PIV, and specifically HALP scores, which are easy to obtain and easy to use in evaluating inflammation, may also be useful in assessing inflammation in *Demodex* blepharitis. Particularly, HALP scores may give clinicians the information about poor immune conditions and chronic inflammation in the patients.

## 1 Introduction

Blepharitis is characterized by ocular irritation, discharge, erythema at the eyelid margin, and rashes on the eyelashes and eyelids [1,2]. In advanced clinical stages, corneal involvement may also be added, and epithelial erosions or keratitis may be observed. Blepharitis has various etiologies, including bacterial, allergic, or seborrheic. *Demodex* mite infestation is one of the most prevalent causes of blepharitis [3].

*Demodex* is a genus of mites that lives in or around hair follicles, sebaceous glands, or the skin. *Demodex* mite is a parasite that survives within the natural and adaptive immune system capabilities of the human skin microflora [3]. It is found in pilosebaceous follicles in the eyelid (more common in the lower eyelid), sebaceous glands, eyelashes, and eyebrows. While demodex mites are not found in newborns, their numbers gradually increase on the skin in infancy, childhood, adolescence, and adulthood [4]. *Demodex* mites contribute to blepharitis by causing direct damage or serving as a vector for *Staphylococcus epidermidis, Staphylococcus aureus,* or *Bacillus oleronius*, causing hypersensitivity and inflammation [5]. However, the pathogenesis of the disease has still not been fully explained. The base of the eyelash follicle is surrounded by a cylindrical collar formed by solidified exudative excretions and called collarettes. In *Demodex* blepharitis, these collarettes are considered pathognomonic [6].

In a suitable microenvironment, *Demodex* mites survive in balance with the human immune system. *Demodex* mite proliferation is inhibited by the human immune system, keeping numbers under control without inducing an inflammatory reaction or any clinical symptoms. This balancing system consists of the microenvironment, *Demodex* mites, and the human immunity system [7]. Whenever this equilibrium changes, it can cause clinical symptoms. The presence of clinical symptoms together with an increase in demodex mite characteristics is called “demodicosis” [8].

An accelerated response to infection to limit the spread of the pathogen is one of the primary functions of inflammation. It has been reported that platelets promote defense mechanisms against microorganisms, and inflammation mediated by platelets has been proven in many infections [9]. In humans and animal models, coagulopathy and thrombosis can be observed following localized and disseminated infections [10].

Recently, new inflammatory markers, including leukocyte subgroups and platelets, the systemic immune inflammation index (SII), and pan–immune–inflammation value (PIV), provide an extensive evaluation of systemic inflammatory status and have been related to poor prognosis in many diseases and infections [11,12,13]. However, we could not find any other study in the literature examining the diagnostic and prognostic values of these biomarkers in *Demodex* mite infestations.

A novel immune-nutritional marker called the hemoglobin, albumin, lymphocytes, and platelet score (HALP) combines routinely collected four indicators of nutritional and immune status as the lymphocyte, platelet, albumin, and hemoglobin [14]. In the literature, HALP has emerged as a new prognostic biomarker of various clinical outcomes. HALP has been studied in acute ischemic stroke, acute heart failure, sleeve gastrectomy, malignancies, and antineutrophil cytoplasmic antibody-associated vasculitis [14,15,16,17,18,19,20]. However, there are a few studies on the relationship between the HALP score and infections [21,22]. Additionally, there are no studies yet showing whether the HALP score is related to *Demodex* infestation. The purpose of our study is to investigate the predictive value and correlation with *Demodex* density and inflammation markers such as CRP, SII, PIV, and HALP scores in patients with *Demodex* blepharitis as a first in the literature.

## 2 Materials and methods

This study was approved by the The Ministry of Health, University Kayseri City Training and Research Hospital Clinical Research Ethics Committee (313/11.02.2025). This study was a retrospective, single-hospital-based, case–control study. It was conducted in compliance with the principles of the Declaration of Helsinki. We enrolled patients with *Demodex* blepharitis who were admitted to our hospital ophthalmology outpatient clinics between January 2023 and September 2024. They were admitted with complaints of crustiness, redness, or itching of the eyelids or symptoms including dry eyes, irritation of the eyes, a foreign body sensation, and a feeling of burning or tearing. The diagnosis of *Demodex* blepharitis was based on microscopic examination of the eyelash samples of 128 patients. Thirty-nine of them had no biochemical or hematological test results and were excluded from the study. The final study population included 89 patients (48 males, 41 females; mean age 54.8 ± 13.76 years), and the control group consisted of 76 individuals (44 males, 32 females; mean age 54.32 ± 7.80 years) without blepharitis. Patients with severe anemia, active autoimmune diseases, malignancy, recent trauma, major surgery, vaccination, infections, or chronic inflammatory diseases that may affect the complete blood count parameters and albumin levels were excluded from the study. Patients who have received blepharitis treatment in the last three months, patients who have used systemic or topical antibiotics or immunosuppressants for any reason, those who have conjunctival diseases such as pterygium and symblepharon that may affect conjunctival colonization, those who have chemical injuries that may affect the ocular surface, acne rosacea, ocular pemphigoid, Stevens–Johnson syndrome and keratoconjunctivitis sicca, mentally retarded patients, contact lens wearers, and patients with atopy and sebaceous dermatitis were excluded. Patients with *Demodex* blepharitis whose medical records were checked retrospectively and had no laboratory test results were also excluded.

### 2.1 Laboratory assay

Laboratory tests, including plasma hemoglobin, lymphocyte, platelet, and serum albumin levels, were analyzed. The complete blood count parameters were analyzed using an autoanalyzer (Sysmex XN-1000 Hematology Analyzer LH750, USA). Serum albumin and CRP levels were assayed using an automatic biochemistry analyzer (Roche COBAS 8000, Switzerland). The method developed by Chen et al. for the HALP score was used to calculate the HALP score as follows: HALP score = [hemoglobin (g/L) × albumin (g/L) × lymphocytes (×10^9^/L)]/platelets (×10^9^/L) [22].

### 2.2 Demodex investigation

A total of 128 patients having blepharitis and the waste product of the *Demodex* based on their eyelashes, called collarettes, were thought to have *Demodex* blepharitis and included in the study. Collarettes are composed of exudative excretions consisting of *Demodex* mites, mite eggs, egg casings, and keratinized cells. Patients were directed to the central laboratory of our hospital, and two eyelash samples were taken from both upper eyelids and lower eyelids by the epilation method. Sampling was done, specifically from eyelashes with intense blepharitis and cylindrical dandruff. The recorded samples were placed directly on the coverslip, glycerol was dropped, the coverslips were covered, and examined under a light microscope as quickly as possible. One or more live *Demodex* mites in the area are examined under a light microscope at 10× magnification is considered as *Demodex* positive.

### 2.3 Statistical analysis

All statistical analyses were performed using SPSS version 23 (SPSS Inc., Chicago, IL, USA). The Kolmogorov–Smirnov and Shapiro–Wilk tests were used to analyze the normal distribution of the variables. To compare the continuous variables with normal distribution, Student’s *t*-test was used; without normal distribution, the Mann–Whitney U test was used. The continuous variables with normal distribution are presented as mean ± standard deviation, and those without normal distribution are presented as median (minimum-maximum). The categorical variables are presented as percentages. The Chi-square test and Fisher’s exact test were performed for comparison of the categorical data. A *p*-value <0.05 was accepted as statistically significant.


**Consent to Participate declaration:** Our study was retrospectively designed, and therefore, there was no need to obtain informed consent to participate approval from the patients.
**Ethics Committee Approval:** The Ministry of Health University Kayseri City Training and Research Hospital Clinical Research Ethics Committee granted approval for this study (313/11.02.2025).

## 3 Results

Among 128 patients with *Demodex* blepharitis, our final study population included 89 patients with demodicidosis and 76 controls without blepharitis. The clinical and laboratory parameters of the groups are presented in Table 1.

**Table 1:** Baseline clinical and laboratory parameters of the study groups

Clinical and laboratory characteristics	Patients	Controls	*p*-value
Age (years)	54.8 ± 13.76	54.32 ± 7.80	0.998
Female (*n*%)	48	44	—
Male (*n*%)	41	32	—
Number of *Demodex* mites	6 ± 2.38	—	—
WBC (×10^9^/L)	7.60 ± 2.08	7.51 ± 2.13	0.862
Hemoglobin (g/dL)	13.34 ± 1.41	14.09 ± 1.58	0.029*
Platelet (×10^9^/L)	284.41 ± 70.76	258.88 ± 52.51	0.096
Neutrophil (×10^9^/L)	4.28 ± 1.33	4.37 ± 1.56	0.709
Lymphocyte (×10^9^/L)	2.59 ± 0.67	2.48 ± 0.72	0.797
Monocyte (×10^9^/L)	0.53 ± 0.16	0.51 ± 0.14	0.440
CRP (mg/L)	4.2 (3.06–6.1)	3.49 (2.45–4.75)	0.174
Albumin (g/L)	42.6 ± 4.2	43.5 ± 3.2	0.266
SII	496.87 (384.79–634.91)	477.56 (328.72–619.30)	0.134
PIV	295.74 (177.59–483.45)	288.36 (171.07–415.40)	0.319
HALP score	437.1 ± 134.9	567.1 ± 194.3	0.001**

WBC, white blood cell; CRP, C-reactive protein; SII, systemic immune inflammation index; PIV, pan–immune–inflammation value; HALP, hemoglobin, albumin, lymphocyte, platelet score. Student’s *t*-test was used for group comparison; data are summarized as mean ± SD. Mann-Whitney U test was used; data are summarized as median and interquartile range (25–75%). *p < 0.05, *p* < 0.001** statistically significant.

These two groups were compared in terms of age, gender, and biochemical and hematological test parameters such as hemoglobin, albumin, neutrophils, lymphocytes, monocytes, platelets, and ratios as HALP score, PIV, systemic immune–inflammation–index (SII), and *Demodex* density (Table 1).

**Figure 1:**
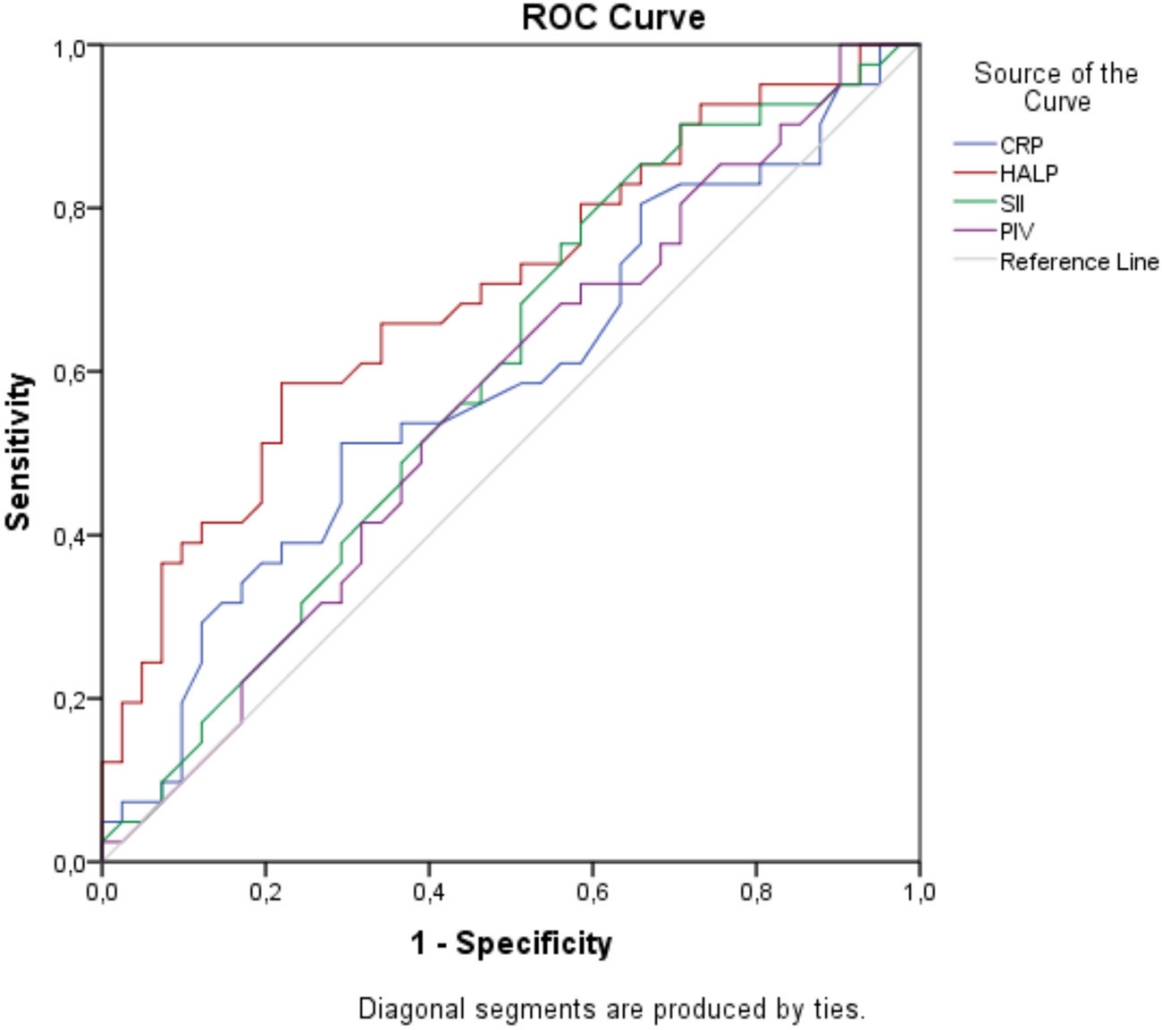
ROC curve analysis of inflammation markers in *Demodex* blepharitis.

Complete blood count parameters, including lymphocytes, neutrophils, monocytes, platelets, and WBC levels, were measured in *Demodex* blepharitis patients and found to be numerically higher but statistically not significant than those of the controls (*p* > 0.05). Whereas, in the patient group, mean hemoglobin levels were statistically significantly lower when compared with the control group (*p* = 0.029). Among the biochemical parameters of patients, CRP values were found to be numerically higher and albumin levels were lower; however, there was no statistically significant difference between these levels in both groups (*p* > 0.05). SII and PIV indices of patients, which were the biomarkers of inflammation calculated by their formulae, were also numerically higher, and HALP score levels were statistically significantly lower when compared with the controls (*p* = 0.134, *p* = 0.319, and *p* = 0.001, respectively; Table 1). ROC analysis was performed, and the curves were drawn to determine the diagnostic performances of inflammatory markers in *Demodex* blepharitis. The areas under the curves were compared (Table 2). It was found that lower HALP score levels were statistically significantly remarkable than higher SII and PIV values in determining *Demodex* blepharitis patients. Then, the optimal cutoff point for HALP scores was detected by the Youden’s index (sensitivity + specificity − 1). It was predicted that values below 504.8 for HALP scores can be used in the diagnosis of *Demodex* blepharitis patients with 66.5% sensitivity and 78% specificity (Table 2, Figure 1).

**Table 2:** ROC curve analysis of inflammation markers

Test result variable(s)	Area under the curve	Std. error	*p*-value	Asymptotic 95% confidence interval	
				Lower bound	Upper bound
CRP	0.587	0.063	0.174	0.463	0.711
HALP	0.697	0.058	0.002	0.584	0.810
SII	0.596	0.063	0.134	0.473	0.720
PIV	0.564	0.064	0.319	0.439	0.689

CRP, C-reactive protein; SII, systemic immune inflammation index; PIV, pan–immune–inflammation value; and HALP, hemoglobin, albumin, lymphocyte, platelet score.

## 4 Discussion

There are two subtypes of *Demodex* in humans: *D folliculorum* and *D brevis*. These mites have no excretory organs, so they regurgitate and combine the undigested material with their eggs, keratin, and epithelial cells of the host. Thus, the bulk of the cylindrical lash deposits are formed that are pathognomonic for *Demodex* infestation. These deposits include lipases and proteases that cause irritation symptoms. Of all the eye symptoms evaluated, the only symptom directly related to Demodex is usually eyelid irritation. These mites’ main food source is sebum, and they use lipolytic enzymes to digest sebum. Therefore, this eyelid irritation is caused by both these lipolytic enzymes and the direct bite of mites [23].

The presence of *Demodex* mites in eyelid tissue and its relationship with blepharitis was established nearly a century ago, but in the past 5–10 years, articles on *Demodex* blepharitis have increased dramatically [1,24]. However, there is still not enough research on the pathogenesis of the disease, which has not been fully explained.

Parasites secrete local immunosuppressive factors for their survival, and the growth of parasites can be enhanced by this immunosuppression. It has been proven that antigens of *Demodex* mites can initiate lymphocyte proliferation [25]. *Demodex* mites also showed to regulate the Toll‐like receptor (TLR) signaling pathway of the human sebocytes, and the immune response of these cells can be affected by bioactive molecules of these mites. Increased mite numbers affect the secretion of interleukin-8 by these cells [26]. However, these inflammation markers are not readily available or routinely investigated markers in all health institutions and hospitals.

In healthy skin, the number of parasites is limited by effective natural immunity. If there is not an adequate immune response against the mite, their number increases, and there are dozens in one hair follicle. When a sufficient immune response cannot be formed against the mite in the presence of a specific genetically determined immune defect or general acquired immune depression, the number of mites increases, and demodex proliferation and demodicosis occur.

*Demodex* mites play a major role in many eye diseases, such as dry eye, meibomian gland dysfunction, chalazion, madarosis (loss of eyelashes), eyelid dermatitis, and blepharitis [27]. The inflammation plays the major role in all these clinical outcomes. However, in the literature, this is the first work evaluating HALP scores, SII, and PIV levels, and comparing the diagnostic performances of these parameters in *Demodex* blepharitis patients. In addition, the diagnostic performance of HALP scores in these patients has been shown to be more significant and higher than WBC and CRP, in addition to SII and PIV values.

*Demodex* blepharitis impairs visual quality and eye comfort, and this has a significant negative impact on the quality of life. *Demodex* can also contribute to corneal damage and ocular surface disorders that can blur visual quality. It can lead to contact lens intolerance or suboptimal surgical outcomes. *Demodex* blepharitis is likely to be an underdiagnosed, significant public health burden, causing patients to visit health care providers multiple times and receive unsuccessful treatment results. Clinical symptoms induced by inflammation cause chronic complaints in patients. In conclusion, our study indicated that inflammation is both the reason and result of blepharitis caused by *Demodex* mites. Therefore, clinicians should be more attentive in routinely examining their patients for *Demodex* infestation and signs of blepharitis and in the best treat of these infestations. In this study, it was found that CRP, SII, PIV, and specifically HALP score values, which are easy to use in assessing the inflammation and easy to achieve, may also be useful in evaluating inflammation in *Demodex* blepharitis. Particularly, HALP scores may give clinicians the information about poor immune conditions and chronic inflammation in these patients.

Since our study was a single-center study with relatively small study groups and a retrospective study, our results should be supported with prospective further studies, including more participants.
